# Malaria, Oromia Regional State, Ethiopia, 2001–2006

**DOI:** 10.3201/eid1707.100942

**Published:** 2011-07

**Authors:** Dereje Olana, Sheleme Chibsa, Dawit Teshome, Addis Mekasha, Patricia M. Graves, Richard Reithinger

**Affiliations:** Author affiliations: Oromia Regional Health Bureau, Addis Ababa, Ethiopia (D. Olana, S, Chibsa, D. Teshome, A. Mekasha);; World Health Organization, Addis Ababa (D. Olana);; US Agency for International Development, Addis Ababa (S. Chibsa, R. Reithinger);; The Carter Center, Atlanta, Georgia, USA (P.M. Graves)

**Keywords:** malaria, parasites, Plasmodium falciparum, Ethiopia, prevention, control, letter

**To the Editor:** In Ethiopia, malaria is unstable and commonly occurs as intraannual and interannual epidemics. Transmission is associated with altitude, temperature, and rainfall, generally peaking twice a year, after the 2 rainy seasons (March–May and July–September) (*1*). Cases are caused by *Plasmodium falciparum* and *P. vivax*. *Anopheles arabiensis* mosquitoes are the main vector for both species. Although malaria is the most common communicable disease in Ethiopia (*2*), few longitudinal case data has been published (*3*).

We report a retrospective analysis of outpatient data for July 2001–June 2006 obtained from all secondary and tertiary government-run health facilities (152 health centers and 25 hospitals) in Oromia Regional State. Oromia has 17 administrative zones and 297 districts. Data were reported monthly on paper forms by health facility staff at district level to the Oromia Regional Health Bureau Zonal Health Offices, which aggregated zonal data before forwarding them to the Oromia Regional Health Bureau Malaria Control Department.

Data obtained included number of outpatient cases (i.e., patients attending the health facility grouped by age <5 years and age >5 years); number of clinical malaria cases (i.e., patients with fever grouped by age and sex); number of clinical cases confirmed by microscopy; and number of cases caused by *P*. *falciparum* and *P*. *vivax.* If no outpatient data were reported, the case number was changed from zero to missing. The data were entered into Microsoft Excel (Microsoft, Redmond, WA, USA) and analyzed by using Stata version 9.0 (StataCorp LP, College Station, TX, USA).

During 2001–2006, a total of 8,786,088 outpatient consultations were reported. A total of 905,467 and 562,996 clinical and confirmed malaria cases, respectively, were reported. Patients were predominantly seen at health centers rather than at hospitals, with 80.2% clinical and 72.2% confirmed malaria cases seen at health centers. Clinical malaria accounted for 10.3% of outpatient consultations in all facilities. However, this percentage varied between years (6.1%–16.0%) and zones (1.3%–21.9%) (Technical Appendix Figure 1).

Of clinical malaria cases, 16.5% were in children <5 years of age (range between years [RBY] 14.0%–18.3%, range between zones [RBZ] 10.9%–61.0%) and 54.3% were in male patients (RBY 52.2%–55.6%, RBZ 50.1%–66.8%). Of clinical malaria cases, 49.2% were confirmed by microscopy (RBY 37.1%–58.0%, RBZ 15.3%–98.4%), and 58.5% (RBY 46.4%–63.4%, RBZ 12.1%–82.4%), and 41.2% (RBY 36.3%–53.4%, RBZ 17.6%–87.9%) of confirmed cases were caused by *P*. *falciparum* and *P*. *vivax*, respectively. Of confirmed cases, 0.4% were caused by mixed *Plasmodium* infections (RBY 0.2%–0.5%, RBZ 0.0%–1.1%). The average incidence of clinical malaria per 100,000 population per month ranged from 14 in February 2002 to 122 in November 2003, and there was considerable variation between months, years, and administrative zones (Technical Appendix Figure 2).

We found that up to 29.0% of outpatient visits to health facilities in certain administrative zones during high transmission years were for malaria. The incidence of malaria is likely to be underestimated because only ≈30% of the population accessed health facilities at that time (*4*). There appeared to be only 1 annual peak of transmission in September–January (Technical Appendix Figure 1). Clinical and confirmed disease varied between zones; 5 of the 15 zones in Oromia (East Hararge, East Shoa, East Wellega, Jimma, West Hararge) reported >75% of the clinical cases seen at health facilities during 2001–2006. Malaria incidence varied between years: clinical and confirmed cases increased in 2003, the last epidemic year recorded in Oromia (*5*), before decreasing to 2001 levels in 2004 (Technical Appendix Figure 1).

The *P*. *falciparum* to *P*. *vixax* ratio changed geographically and temporally (Technical Appendix Figure 1), and increases in the proportion of *P*. *falciparum* cases coincided with the peak malaria transmission season. In the epidemic year of 2003, the proportion of *P*. *falciparum* cases was larger than in other years, and children <5 years of age were disproportionately affected (Technical Appendix Figure 1). Contrary to previous reports (*6*), our data did not indicate a change in the *P*. *falciparum* to *P*. *vivax* ratio after artemether/lumefantrine was introduced in 2005.

Health facility data can have many caveats (*7*), including concerns about data representativeness (e.g., if only a small number of facilities are assessed); data validity, particularly if, as was the case during that time, only limited diagnostic quality assurance was available (*8*); and analytical approaches used. Our analysis comprised all Oromia secondary and tertiary facilities; only 3.4% of health centers and 13.0% of hospitals surveyed had no data, suggesting that given the extensive data reported, these missing data would have only marginally affected the temporal and spatial trends observed.

Our data complement those of recent cross-sectional surveys (*9*) and provide a useful baseline to assess scale-up of malaria prevention and control efforts. Unlike cross-sectional and small-scale facility surveys (*6*), our comprehensive longitudinal monthly data monitored disease trends spatially and temporally, showing that malaria still represented a major health services problem until 2006.

## Supplementary Material

Technical Appendix Outpatient, clinical, and parasitologically confirmed malaria in Oromia Regional State, Ethiopia, 2001-2006. Figure 1. Number of total clinical malaria cases (bars) and cases in children <5 years of age (thick line), proportion of clinical malaria cases confirmed parasitologically (dashed line), and proportion of confirmed malaria cases caused by Plasmodium falciparum (solid line). Figure 2. Annual incidence of clinical malaria cases/100,000 population in individual administrative zones (gray lines) and average malaria incidence (thick line).
