# Supplementary material for: Malaria, Oromia Regional State, Ethiopia, 2001–2006
Source: Emerg Infect Dis. 2011 Jul;17(7):1336–7. doi: 10.3201/eid1707.100942 (PMC3381418; doi:10.3201/eid1707.100942)
Supplement: Technical Appendix — Outpatient, clinical, and parasitologically confirmed malaria in Oromia Regional State, Ethiopia, 2001-2006. Figure 1. Number of total clinical malaria cases (bars) and cases in children <5 years of age (thick line), proportion of clinical malaria cases confirmed parasitologically (dashed line), and proportion of confirmed malaria cases caused by Plasmodium falciparum (solid line). Figure 2. Annual incidence of clinical malaria cases/100,000 population in individual administrative zones (gray lines) and average malaria incidence (thick line). [file 10-0942-Techapp.pdf]

Article DOI: 10.3201/eid1707.100942

Malaria, Oromia Regional State, Ethiopia, 2001–2006

Technical Appendix

Outpatient, Clinical, and Parasitologically Confirmed Malaria

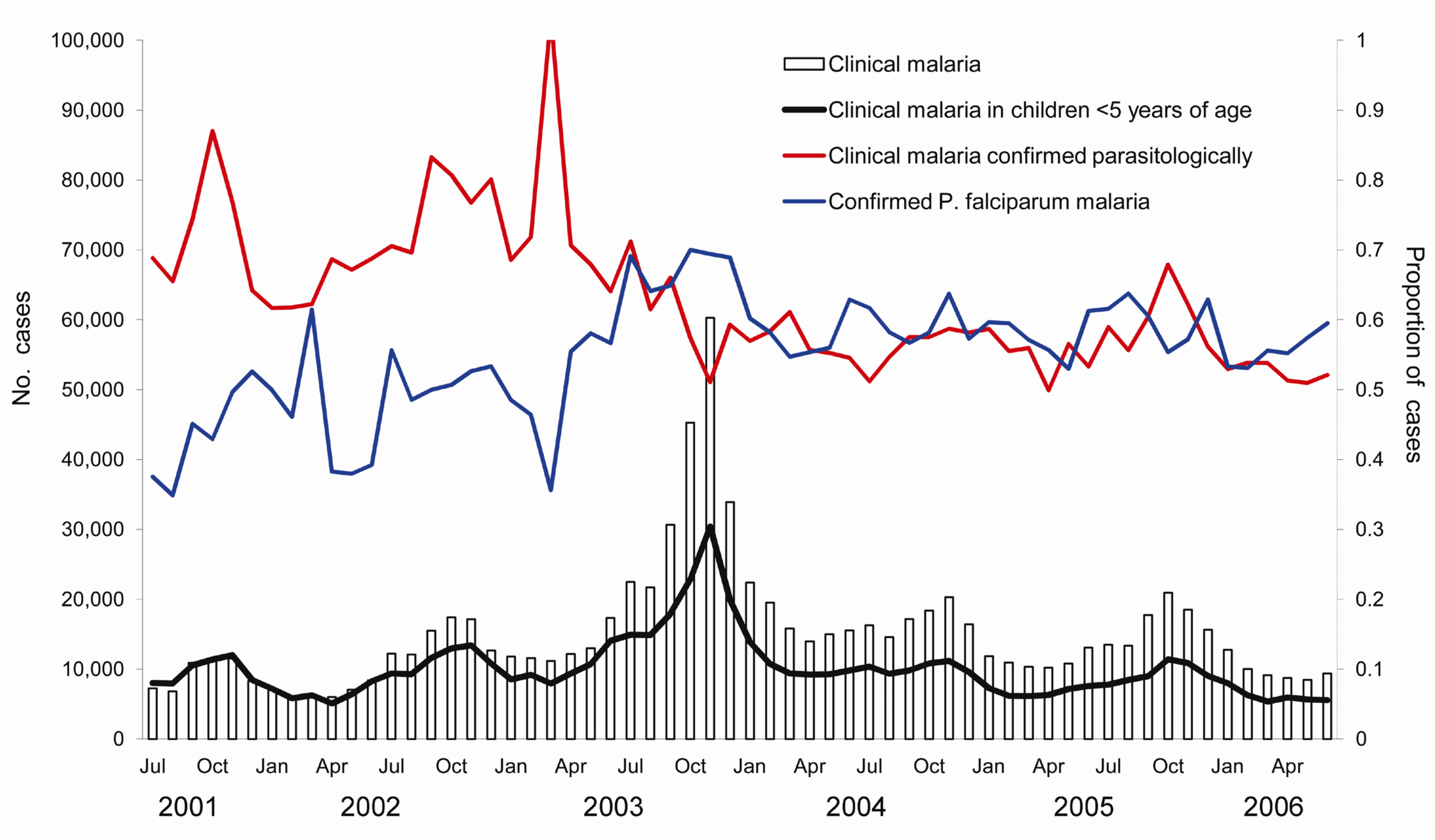

Figure 1. Number of total clinical malaria cases (bars) and cases in children <5 years of age (black line), proportion of clinical malaria cases confirmed parasitologically (red line), and proportion of confirmed malaria cases caused by *Plasmodium falciparum* (blue line).

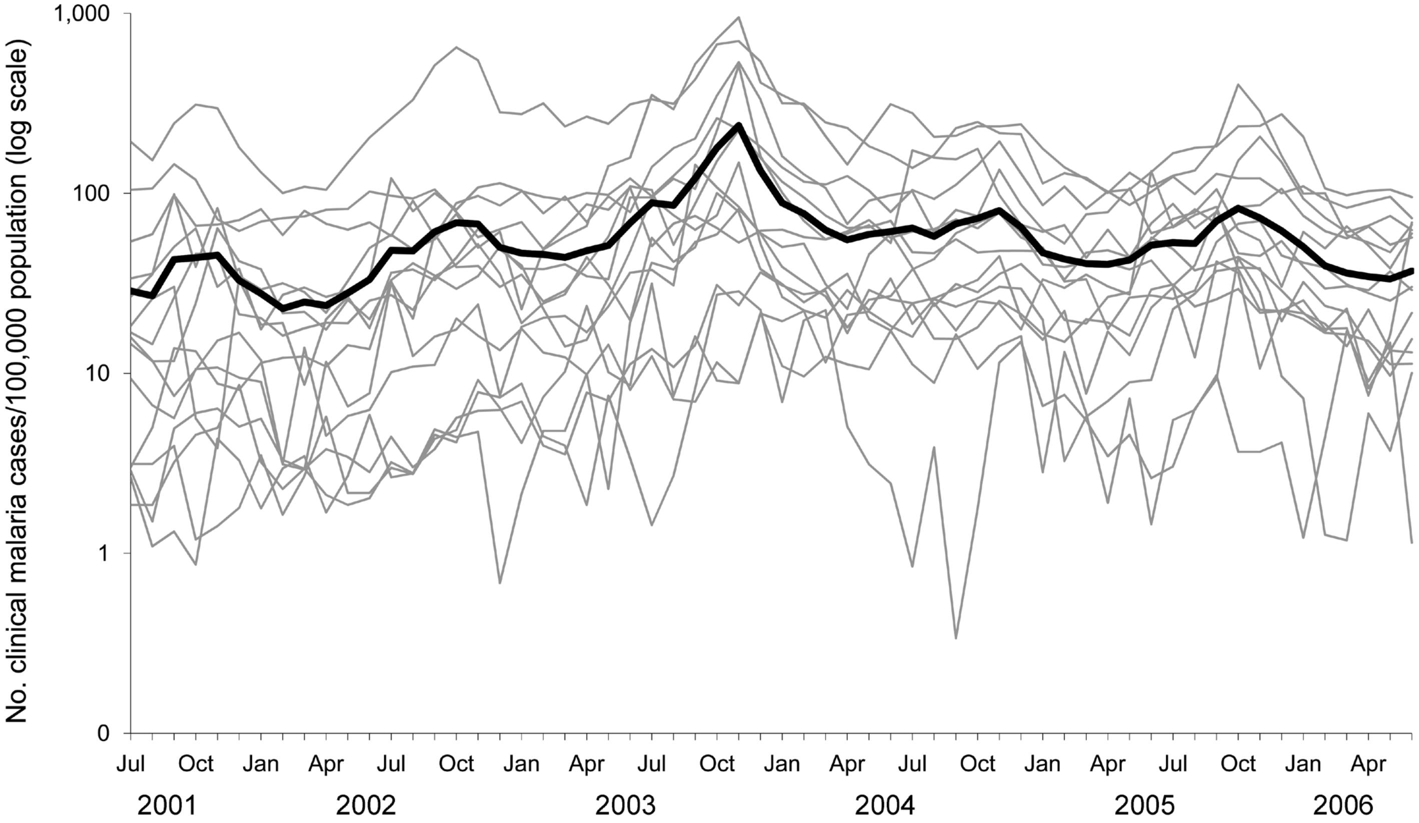

Figure 2. Annual incidence of clinical malaria cases/100,000 population in individual administrative zones (gray lines) and average malaria incidence (black line).
